# Efficient and Secure Temporal Credential-Based Authenticated Key Agreement Using Extended Chaotic Maps for Wireless Sensor Networks

**DOI:** 10.3390/s150714960

**Published:** 2015-06-25

**Authors:** Tian-Fu Lee

**Keywords:** authentication, privacy protection, key agreement, temporal credential, wireless sensor networks, chaotic maps

## Abstract

A secure temporal credential-based authenticated key agreement scheme for Wireless Sensor Networks (WSNs) enables a user, a sensor node and a gateway node to realize mutual authentication using temporal credentials. The user and the sensor node then negotiate a common secret key with the help of the gateway node, and establish a secure and authenticated channel using this common secret key. To increase efficiency, recent temporal credential-based authenticated key agreement schemes for WSNs have been designed to involve few computational operations, such as hash and exclusive-or operations. However, these schemes cannot protect the privacy of users and withstand possible attacks. This work develops a novel temporal credential-based authenticated key agreement scheme for WSNs using extended chaotic maps, in which operations are more efficient than modular exponential computations and scalar multiplications on an elliptic curve. The proposed scheme not only provides higher security and efficiency than related schemes, but also resolves their weaknesses.

## 1. Introduction

Wireless sensor networks (WSNs) comprise a large number of sensor nodes, and are utilized in many environments, such as dangerous areas in which humans must be medically monitored, military environments in which reconnaissance and communication must be carried out, and others. Owing to the hardware limitations, sensor nodes in WSNs cannot support heavy computation loads, extensive communications or extensive storage. Thus, developing a lightweight and secure authenticated key agreement scheme is very important for WSNs. Temporal credential-based authenticated key agreements enable communicating entities to authenticate each other and to establish a secure and authenticated channel by confirming their temporal credentials. A temporal credential-based authenticated key agreement scheme for WSNs is composed of three classes of entity—users, sensor nodes and a gateway node (*GWN*)—and has registration, login, authentication and key agreement, and password change phases. In the registration phase, users and sensor nodes register their secret keys to the *GWN*. Then the *GWN* issues one temporal credential to each user and sensor node for authentication. In the login, authentication and key agreement phases, the user, the sensor node and *GWN* authenticate each other using these temporal credentials. Additionally, the user and the each sensor node negotiate a common secret key with the help of *GWN* to establish a secure and authentication channel in the WSN. Finally, the password change phase enables users to update their passwords for increased security [1,2,3,4,5,6,7,8,9].

Recently, Xue *et al.* [8] presented the concept of temporal credentials and developed a lightweight temporal credential-based authenticated key agreement scheme for WSNs. The scheme of Xue *et al.* has a lower computational burden, less extensive communication needs and requires less storage than previous approaches, and tries to provide more functionality and higher security [10,11,12,13,14,15,16,17]. Later, Li *et al.* [9] noted that the scheme of Xue *et al.* fails to withstand stolen-verifier attacks, password guessing attacks, insider attacks and lost smartcard attacks, and so proposed an advanced temporal credential-based scheme for WSNs as an alternative. However, in the scheme of Li *et al.*, an adversary can derive users’ identities, temporal credentials, verification values in the *GWN*’s verifier table and expiration time from revealed messages allowing the adversary to perform successful impersonation attacks and stolen verifier attacks, easily discovering the hidden identity of the sender of the request message. Moreover, the adversary can derive all previous session keys of users and sensor nodes, and thus access all transmitted secrets. Accordingly, these temporal credential-based schemes for WSNs fail to resist possible attacks and to protect the privacy of users.

### 1.1. Our Contributions

This work addresses the weaknesses of the scheme of Li *et al.* and proposes an efficient and secure temporal credential-based authenticated key agreement scheme for WSNs that uses extended chaotic maps, and involves operations that are more efficient than modular exponential computations and scalar multiplications on an elliptic curve [18,19,20]. The proposed scheme protects a user’s identity using a temporary secret key of the user and the gateway node, which security is based on the extended chaotic maps-based Diffie-Hellman problem [21,22,23,24,25,26,27], and reduces the number of parameters concerning each user’s identity and password such that an adversary cannot impersonate any user or communicate with the gateway node or the sensor nodes, even if the adversary has stolen the verifier table and obtained the user’s private information. Additionally the ephemeral parameters are randomly selected and independent among executions of the scheme. Thus, the adversary cannot derive any previous session keys of the user and the sensor node. The proposed scheme avoids the weaknesses of previous schemes, has higher security and lower computational cost.

### 1.2. Enhanced Chebyshev Polynomial and Extended Chaotic Maps

Recent investigations have demonstrated that cryptosystems that use chaotic map operations are more efficient than those that use modular exponential computations and scalar multiplications on elliptic curves. Additionally, enhanced Chebyshev polynomials also exhibit the semi-group property and the commutative property, and they are subject to the discrete logarithm problem and the Diffie-Hellman problem [21,22,23,24,25,26,27], which are described as follows.

#### 1.2.1. Enhanced Chebyshev Polynomial

The enhanced Chebyshev polynomial *T_n_*(*x*) is a polynomial in *x* of degree *n*, defined by the following recurrence relation:

(1) $$\left\{ \begin{array}{l} T_{0}(x)=1\\ T_{1}(x)=x\text{; and}\\ T_{n}(x)=2xT_{n-1}(x)-T_{n-2}(x)\text{mod}\;p\text{, for}\;n\geq 2 \end{array}\right.$$

where and *p* is a large prime number. The enhanced Chebyshev polynomials satisfy the semi-group property and are commutative under composition. Then:

*T_r_*(*T_s_*(*x*)) ≡ *T_rs_*(*x*) ≡ *T_s_*(*T_r_*(*x*)) mod *p* (2)

holds.

#### 1.2.2. Extended Chaotic Map-Based Discrete Logarithm Problem

Given *x*, *y* and *p*, it is computationally infeasible to find the integer *r* satisfying:

*y = T_r_*(*x*) mod *p* (3)

#### 1.2.3. Extended Chaotic Map-Based Diffie-Hellman Problem

Given *T_u_*(*x*), *T_v_*(*x*), *T*(.), *x* and *p*, where *u*, *v* ≥ 2, *x*∈(−∞, +∞) and *p* is a large prime number, it is computationally infeasible to calculate:

*T_u⋅v_*(*x*) ≡ *T_u_*(*T_v_*(*x*)) ≡ *T_v_*(*T_u_*(*x*)) mod *p* (4)

### 1.3. Organization of the Paper

The rest of this paper is organized as follows: Section 2 reviews the temporal credential-based scheme of Li *et al.* for WNSs and elucidates its weaknesses. Section 3 presents the proposed efficient and secure temporal credential-based authenticated key agreement scheme for WSNs using extended chaotic maps. Section 4 and Section 5 present the results of evaluations of the security and performance of the scheme, respectively. Finally, Section 6 draws conclusions.

## 2. The Temporal Credential-Based Scheme of Li *et al.* and Its Weaknesses

This section presents the notation used in this study, briefly reviews the advanced temporal credential-based scheme for wireless sensor networks proposed by Li *et al.* [9], and finally states its weaknesses.

Assume that *U_i_* denotes the *i-*th user of WSNs; *S_j_* denotes the *j-*th sensor node; and *GWN* denotes the Gateway node in which *U_i_* and *S_j_* are registered. Table 1 lists the notations which are used throughout this paper.

**Table 1:** Notation.

*ID_i_, PW_i_*	Identity and password pair of user *U_i_*
*SID_j_*	Pre-configured identity of the sensor node *S_j_*
*K_GWN_U_, K_GWN_S_*	The long-term secret keys only known to *GWN*.
*p*	A large prime number
*TCR_i_, TCR_j_*	A temporal credential issued by *GWN* to *U_i_* / *S_j_*
*E_i_*	The expiration time of *U_i_*’s temporal credential.
*t*_1_,*t*_2_,…,*t*_6_	The timestamp values.
Δ*t*	The expected time interval for the transmission delay.
*h*(.)	A collision free one-way hash function [28]
*A*→*B:M*	A sends message *M* to *B* through a common channel.
⊕	The exclusive-or (XOR) operation
*M*_1_||*M*_2_	Message *M*_1_ concatenates to message *M*_2_.

### 2.1. Review of the Temporal Credential-Based Scheme of Li et al.

In 2013, Li *et al.* [9] proposed an advanced temporal credential-based scheme for WSNs, which consists of pre-registration, registration, login, authentication and key agreement phases, which are described as follows.

#### 2.1.1. Pre-Registration Phase

Each user *U_i_* has a pair of identity *ID^pre^_i_* and password *PW^pre^_i_*. *GWN* stores *h*(*ID^pre^_i_*||*PW^pre^_i_*) and *ID^pre^_i_* in its storage. Similarly, each sensor node *S_j_* is pre-configured with its identity *SID_j_* and a random number *r_j_* and the hash value *h*(*SID_j_*||*r_j_*). Then *r_j_* and *SID_j_* are stored on the *GWN*’s storage. 

#### 2.1.2. Registration Phase

(1) Registration phase for users
  Step 1: *U_i_* → *GWN*: {*ID^pre^_i_*, *t*_1_,*VI_i_*, *CI_i_*, *DI_i_*}
    *U_i_* selects his/her *ID_i_*, password *PW_i_*, and a random number *r_i_*, computes and sends {*ID^pre^_i_*, *t*_1_, *VI_i_*, *CI_i_*, *DI_i_*} to *GWN*, where *VI_i_* = *h*(*t*_1_||*h*(*ID^pre^_i_*||*PW^pre^_i_*)), *CI_i_* = *h*(*ID^pre^_i_*||*PW^pre^_i_*) ⨁ *h*(*ID_i_*||*PW_i_*||*r_i_*), *DI_i_* = *ID_i_* ⨁ *h*(*ID^pre^_i_*||*PW^pre^_i_*) and *t*_1_ is the current timestamp.
  Step 2: *GWN* → *U_i_*: {*h*(*Q_i_*), smartcard}
    *GWN* checks the validity of *t*_1_, retrieves *h*(*ID^pre^_i_*||*PW^pre^_i_*) by using *ID^pre^_i_*, computes VI_i_^*^ = *h*(*t*_1_||*h*(*ID^pre^_i_*||*PW^pre^_i_*)) and checks VI_i_^*^ =? *VI_i_*. Then *GWN* computes *Q_i_* = *CI_i_* ⨁ *h*(*ID^pre^_i_*||*PW^pre^_i_*) = *h*(*ID_i_*||*PW_i_*||*r_i_*), *DI_i_* = *ID_i_* ⨁ *h*(*ID^pre^_i_*||*PW^pre^_i_*), *P_i_* = *h*(*ID_i_*||*E_i_*), *TCR_i_* = *h*(*K_GMN_U_*||*P_i_*||*E_i_*) and *PTC_i_* = *TCR_i_* ⨁ *Q_i_* and personalizes the smart card for *U_i_* with the parameters: {*h*(.), *h*(*Q_i_*), *E_i_*, *PTC_i_*}. *GWN* maintains a write protected file, where the status-bit indicates the status of the user, *i.e.*, when *U_i_* is logged-in to *GWN*, the status-bit is 1, otherwise it is 0. Finally, *GWN* sends *h*(*Q_i_*) and smart card to *U_i_*.
  Step 3: *U_i_* and authenticates *GWN* by checking *h*(*h*(*ID_i_*||*PW_i_*||*r_i_*)) =? *h*(*Q_i_*) and enters *r_i_* into his/her smart card. Then the smart card contains {*h*(.), *h*(*Q_i_*), *E_i_*, *PTC_i_*, *r_i_*}.
(2) Registration phase for sensor nodes
  Step 1: *S_j_* → *GWN*: {*SID_j_*, *t*_2_,*VI_j_*}
    *S_j_* computes *VI_j_* = *h*(*t*_2_||*h*(*SID_j_*||*r_j_*)) and sends {*SID_j_*, *t*_2_,*VI_j_*} to *GWN*, where *t*_2_ is the current timestamp.
  Step 2: *GWN* → *S_j_*: {*t*_3_, *Q_j_*, *REG_j_*}
    *GWN* checks the validity of *t*_2_, retrieves *h*(*SID_j_*||*r_j_*) by using *SID_j_* and computes VI_j_^*^ = *h*(*t*_2_||*h*(*SID_j_*||*r_j_*)), checks VI_j_^*^ =? *VI_j_*, computes *TCR_j_* = *h*(*K_GMN_S_*||*SID_j_*), *Q_j_* = *h*(*t*_3_||*h*(*SID_j_*||*r_j_*)) and *REG_j_* = *h*(*h*(*SID_j_*||*r_j_*)||*t*_3_) ⨁ *TCR_j_*, and sends {*t*_3_, *Q_j_*, *REG_j_*} to *S_j_*, where *t*_3_ is the current system timestamp.
  Step 3: *S_j_* checks the validity of *t*_3_ and *h*(*t*_3_||*h*(*SID_j_*||*r_j_*)) =? *Q_j_*, computes its temporal credential *TCR_j_* = *REG_j_* ⨁ *h*(*h*(*SID_j_*||*r_j_*)||*t*_3_) and stores it.

#### 2.1.3. Login Phase

Step 1: *U_i_* inserts his/her smart card into a card reader and enters *ID_i_* and *PW_i_*.
Step 2: The smartcard retrieves *r_i_*, computes *Q_i_'* = *h*(*ID_i_*||*PW_i_*||*r_i_*) and checks *h*(*Q_i_'*) =? *h*(*Q_i_*). If successful, *U_i_* passes the verification, allows to read the information stored in the smartcard, and computes *TCR_i_* = *PTC_i_* ⨁ *Q_i_'*.

#### 2.1.4. Authentication and Key Agreement Phase

Step 1: *U_i_* → *GWN*: {*DID_i_*, *C_i_*, *PKS_i_*, *t*_4_, *E_i_*, *P_i_*}
  *U_i_* computes *DID_i_* = *ID_i_* ⨁ *h*(*TCR_i_*||*t*_4_), *C_i_* = *h*(*h*(*ID_i_*||*PW_i_*||*r_i_*)||*t*_4_) ⨁ *TCR_i_*, *PKS_i_* = *K_i_* ⨁ *h*(*TCR_i_*||*t*_4_||"000"), and sends {*DID_i_*, *C_i_*, *PKS_i_*, *t*_4_, *E_i_*, *P_i_*} to *GWN*, where *t*_4_ is the current timestamp.
Step 2: *GWN* → *S_j_*: {*t*_5_, *DID_i_*, *DID_GWN_*, *C_GWN_*, *PKS_GWN_*}
  *GWN* checks the validity of *t*_4_, computes TCR_i_^*^ = *h*(*K_GMN_U_*||*P_i_*||*E_i_*) and *ID_i_* = *DID_i_* ⨁ *h*(TCR_i_^*^||*t*_4_) and retrieves *U_i_*'s password-verifier of *Q_i_* = *h*(*ID_i_*||*PW_i_*||*r_i_*) by using *ID_i_*. Then, *GWN* further computes C_i_^*^ = *h*(*Q_i_* ||*t*_4_) ⨁ TCR_i_^*^, verifies C_i_^*^ =? *C_i_*, sets the status-bit as “1” and records *t*_4_ in the 4th field of the identity table. *GWN* computes *K_i_* = *PKS_i_* ⨁ *h*(TCR_i_^*^||*t*_4_||"000") and chooses a nearby suitable sensor node *S_j_* as the accessed sensor node. *GWN* further computes *S_j_*’s temporal credential *TCR_j_ = h*(*K_GWN_S_||SID_j_*), *DID_GWN_* = *ID_i_* ⨁ *h*(*DID_i_*||*TCR_j_*||*t*_5_), *C_GWN_* = *h*(*ID_i_*||*TCR_j_*||*t*_5_) and *PKS_GWN_* = *K_i_* ⨁ *h*(*TCR_i_*||*t*_5_) and sends {*t*_5_, *DID_i_*, *DID_GWN_*, *C_GWN_*, *PKS_GWN_*} to *S_j_*, where *t*_5_ is the current timestamp of *GWN*.
Step 3: *S_j_* → *GWN*, *U_i_*: {*SID_j_*, *t*_6_, *C_j_*, *PKS_j_*}
  *S_j_* checks the validity of *t*_5_, computes *ID_i_* = *DID_GWN_* ⨁ *h*(*DID_i_*||*TCR_j_*||*t*_5_) and C_GWN_^*^ = *h*(*ID_i_*||*TCR_j_*||*t*_5_), and checks C_GWN_^*^ =? *C_GWN_*. If unsuccessful, *S_j_* terminates this session; otherwise, *S_j_* convinces that the received message is from a legitimate *GWN*. Moreover, *S_j_* computes *K_i_* = *PKS_GWN_* ⨁ *h*(*TCR_i_*||*t*_5_), *C_j_* = *h*(*K_j_*||*ID_i_*||*SID_j_*||*t*_6_) and *PKS_j_* = *K_j_* ⨁ *h*(*K_i_*||*t*_6_) and sends {*SID_j_*, *t*_6_, *C_j_*, *PKS_j_*} to *GWN* and *U_i_*, where *t*_6_ is the current timestamp of *S_j_*.
Step 4: *U_i_* and *GWN* separately computes *K_j_* = *PKS_j_* ⨁ *h*(*K_i_*||*t*_6_) and C_j_^*^= *h*(*K_j_||ID_i_||SID_j_||t*_6_). *GWN* authenticates *S_j_* by checking C_j_^*^ =? *C_j_*. *U_i_* authenticates *S_j_* and *GWN* by checking C_j_^*^ =? *C_j_*. Finally, *U_i_* and *S_j_* computes a common session key *K_ij_* = *h*(*K_i_*||*K_j_*) for later securing communications.

### 2.2. Weaknesses of Temporal Credential-Based Scheme of Li et al.

This subsection elucidates the weaknesses of the temporal credential-based scheme of Li *et al.*, which include vulnerability to impersonation and stolen verifier attacks, and failure to protect the privacy of users.

#### 2.2.1. Vulnerability to Impersonation Attacks

In the registration phase of the scheme of Li *et al.*, since (*ID^pre^_i_*, *t*_1_, *VI_i_*, *CI_i_*, *DI_i_*) and (*h*(*.*), *h*(*Q_i_*), *E_i_*, *PTC_i_*) are public, where *VI_i_* = *h*(*t*_1_||*h*(*ID^pre^_i_*||*PW^pre^_i_*)), *CI_i_* = *h*(*ID^pre^_i_*||*PW^pre^_i_*) ⨁ *h*(*ID_i_*||*PW_i_*||*r_i_*), *DI_i_* = *ID_i_* ⨁ *h*(*ID^pre^_i_*||*PW^pre^_i_*) and *t*_1_ is the current timestamp, an adversary, A, can obtain a correct *PW^pre^_i_* by guessing a password PW^pre^^*^_i_ and checking *VI_i_* = ? *h*(*t*_1_||*h*(*ID^pre^_i_*||PW^pre^^*^_i_)) repeatedly. Next, the adversary can derive *ID_i_*, *Q_i_* ( =*h*(*ID_i_*||*PW_i_*||*r_i_*) ) and *TCR_i_* by computing *DI_i_* ⨁ *h*(*ID^pre^_i_*||*PW^pre^_i_*), *h*(*ID^pre^_i_*||*PW^pre^_i_*) ⨁ *CI_i_* and *PTC_i_* ⨁ *Q_i_*|, respectively. A can subsequently impersonate *U_i_* and compromise *U_i_*'s privacy based on knowledge of (*ID_i_*, *Q_i_*, *TCR_i_*, *E_i_*). By the following steps, A can successfully impersonate *U_i_*, be authenticated, and communicate with *GWN* and *S_j_*:

Step 1: First, the adversary A retrieves *P_i_* using *E_i_*. In the authentication and key agreement phase, A can compute *DID_i_* =*ID_i_* ⨁ *h*(*TCR_i_*||*t*_4_), *C_i_* = *h*(*h*(*Q_i_*||*t*_4_)⨁*TCR_i_*), *PKS_i_* = *K_i_* ⨁ *h*(*TCR_i_*||*t*_4_||"000"), where *t*_4_ is the current timestamp. Then, A successfully impersonates *U_i_* and sends {*DID_i_*, *C_i_*, *PKS_i_*, *t*_4_, *E_i_*, *P_i_*} to *GWN*.
Step 2: *GWN* checks *t*_4_, computes TCR_i_^*^ =*h*(*K_GWN_U_*||*P_i_*||*E_i_*) and *ID_i_* =*DID_i_* ⨁ *h*(TCR_i_^*^||*t*_4_), C_i_^*^ = *h*(*h*(*Q_i_*||*t*_4_)⨁TCR_i_^*^) and verifies C_i_^*^ =? C_i_^*^. Then, *GWN* computes *K_i_* = *PKS_i_* ⨁ *h*(*TCR_i_*||*t*_4_||"000"), *TCR_j_* =*h*(*K_GWN_S_*||*SID_j_*), *DID_GWN_* = *ID_i_* ⨁ *h*(*DID_i_*||*TCR_j_*||*t*_5_), *C_GWN_* = *h*(*ID_i_*||*TCR_j_*||*t*_5_) and *PKS_GWN_* = *K_i_* ⨁ *h*(*TCR_i_*||*t*_5_) and sends {*t*_5_, *DID_i_*, *DID_GWN_*, *C_GWN_*, *PKS_GWN_*} to *S_j_*, where *t*_5_ is the current timestamp of *GWN*.
Step 3: *S_j_* checks *t*_5_, computes *ID_i_* = *DID_GWN_* ⨁ *h*(*DID_i_*||*TCR_j_*||*t*_5_), C_GWN_^*^ = *h*(*ID_i_*||*TCR_j_*||*t*_5_), *K_i_* = *PKS_GWN_* ⨁ *h*(*TCR_i_*||*t*_5_)and *C_j_* = *h*(*K_j_*||*ID_i_*||*SID_j_*||*t*_6_); verifies C_GWN_^*^ =? *C_GWN_*, and responds by sending {*SID_j_*, *t*_6_, *C_j_*, *PKS_j_*} to *GWN* and A, where *PKS_j_* = *K_j_* ⨁ *h*(*K_i_*||*t*_6_). Finally, A computes *K_j_* = *PKS_j_* ⨁ *h*(*K_i_*||*t*_6_) and shares the common session key *K_ij_* = *h*(*K_i_*||*K_j_*) with *S_j_*.

However, if the password *PW^pre^_i_* is sufficiently long, the credential based key agreement scheme of Li, *et al.* can resist the impersonation attacks.

#### 2.2.2. Failure to Protect the Privacy of Users

In the scheme of Li *et al.*, upon receiving the request message {*DID_i_*, *C_i_*, *PKS_i_*, *t*_4_, *E_i_*, *P_i_*} that is sent by *U_i_*, whose identity is *ID_i_*, the adversary A easily determines that the request message belongs to *U_i_* because A has the knowledge of (*ID_i_*, *Q_i_*, *TCR_i_*, *E_i_*). Thus, the scheme of Li *et al.* fails to support user anonymity, data unlinkability, or untrackability [29]. Accordingly, the scheme of Li *et al.* cannot protect the privacy of users.

#### 2.2.3. Vulnerability to Stolen Verifier Attacks

Assume that an adversary A steals the verifier table and obtains (*ID_i_*, *Q_i_*, *E_i_*). The adversary A can derive *TCR_i_* using *PTC_i_* ⨁ *Q_i_*, since (*h*(*.*), *h*(*Q_i_*), *E_i_*, *PTC_i_*) is public in the registration phase:

Step 1: A → *GWN*: {DID_i_^**^, *C_i_ t*_4_^**^, *PKS_i_*, *t*_4_^**^, *E_i_*, *P_i_*}
  A randomly selects K_i_^**^, computes DID_i_^**^ = *ID_i_* ⨁ *h*(*TCR_i_*||*t*_4_^**^), C_i_^**^ = *h*(*Q_i_*||*t*_4_^**^) ⨁ *TCR_i_* and PKS_i_^**^ = K_i_^**^ ⨁ *h*(*TCR_i_*||*t*_4_^**^||"000"), where *t*_4_^**^ is the current timestamp, and sends {DID_i_^**^, *C_i_ t*_4_^**^, *PKS_i_*, *t*_4_^**^, *E_i_*, *P_i_*} to *GWN*.
Step 2: *GWN* → *S_j_*: {*t*_5_, DID_i_^**^, *DID_GWN_*, *C_GWN_*, *PKS_GWN_*}
  *GWN* validates *t*_4_^**^, computes TCR_i_^*^ = *h*(*K_GMN_U_*||*P_i_*||*E_i_*) and *ID_i_* = DID_i_^**^ ⨁ *h*(TCR_i_^*^||*t*_4_^**^), and retrieves *Q_i_* = *h*(*ID_i_*||*PW_i_*||*r_i_*). Then, *GWN* verifies *h*(*Q_i_* ||*t*_4_^**^) ⨁ TCR_i_^*^ = C_i_^**^, computes *K_i_* = *PKS_i_* ⨁ *h*(TCR_i_^*^||*t*_4_^**^||"000") , *TCR_j_ = h*(*K_GWN_S_||SID_j_*), *DID_GWN_* = *ID_i_* ⨁ *h*(DID_i_^**^||*TCR_j_*||*t*_5_), *C_GWN_* = *h*(*ID_i_*||*TCR_j_*||*t*_5_) and *PKS_GWN_* = *K_i_* ⨁ *h*(*TCR_i_*||*t*_5_), and sends {*t*_5_, DID_i_^**^, *DID_GWN_*, *C_GWN_*, *PKS_GWN_*} to *S_j_*, where *t*_5_ is the current timestamp of *GWN*.
Step 3: *S_j_* → *GWN*, *U_i_*: {*SID_j_*, *t*_6_, *C_j_*, *PKS_j_*}
  *S_j_* validates *t*_5_. If successful, *S_j_* computes *ID_i_* = *DID_GWN_* ⨁ *h*(DID_i_^**^||*TCR_j_*||*t*_5_) and C_GWN_^*^ = *h*(*ID_i_*||*TCR_j_*||*t*_5_) and checks C_GWN_^*^ =? *C_GWN_*, computes K_i_^**^ = *PKS_GWN_* ⨁ *h*(*TCR_i_*||*t*_5_), *C_j_* = *h*(*K_j_*||*ID_i_*||*SID_j_*||*t*_6_) and *PKS_j_* = *K_j_* ⨁ *h*(K_i_^**^||*t*_6_) and sends out {*SID_j_*, *t*_6_, *C_j_*, *PKS_j_*}.
Step 4: Upon receiving {*SID_j_*, *t*_6_, *C_j_*, *PKS_j_*}, A computes *K_j_* = *PKS_j_* ⨁ *h*(K_i_^**^||*t*_6_) and a common session key *K_ij_* = *h*(*K_i_*||*K_j_*) that is shared with *S_j_*.

Hence, the adversary A can impersonate *U_i_*, be authenticated, and communicate with *GWN* and *S_j_*. Additionally, A has *TCR_i_* and messages (*PKS_i_*, *t*_4_) and (*PKS_j_*, *t*_6_), which were previously sent out by user *U_i_*. A can therefore derive previous secrets *K_i_* and *K_j_* by computing *PKS_i_* ⨁ *h*(*TCR_i_*||*t*_4_||"000") and *PKS_i_* ⨁ *h*(*K_i_*||*t*_6_), respectively. A can calculate all session keys that have been used by *U_i_* and *S_j_*, and thereby derive all transmitted secrets. Therefore, the authenticated key agreement scheme of Li *et al.* fails to resist stolen verifier attacks.

## 3. Proposed Temporal Credential-Based Scheme Using Chaotic Maps for WSNs

This section describes the use of chaotic maps in a new temporal credential-based authenticated key agreement scheme for WSNs. The novel scheme does not reveal the user’s private parameters in the registration phase, and it protects the user’s identity with a temporary secret key of the user and the gateway node. The security of this temporary secret key is based on the extended chaotic map-based Diffie-Hellman problem. The proposed approach also reduces the redundant parameters associated with the user’s identity and password, which are stored in the *GWN*’s verifier table, preventing an adversary from impersonating a user and communicating with the gateway node and sensor nodes, even if the adversary has stolen the verifier table and obtained the user’s private information. The session key security is based on the extended chaotic map-based Diffie-Hellman problem, so the adversary cannot derive any previous session key of the user and the sensor node. In the proposed scheme, the user does not know which node it can access and communicate with, thus *GWN* requires choosing a nearby suitable sensor node as the accessed sensor node. The proposed scheme involves parameter generation, pre-registration, registration, login and authentication and password change phases, which are described below.

### 3.1. Parameter Generation Phase

Step 1: The gateway node *GWN* randomly selects *K_GWN_* as its master secret key.
Step 2: *GWN* computes *PK_G_* = *T_K__GWN_*(*x*) mod *p*, where *x* is a random number, *p* is a large prime number and (*PK_G_*, *T*(.), *x*, *p*) are public parameters.

### 3.2. Pre-Registration Phase

Each user *U_i_* has a pre-configured identity *ID^pre^_i_*, which is stored in the *GWN*’s storage. Similarly, each sensor node *S_j_* is pre-configured with its identity *SID_j_* and a random number *r_j_* and the hash value *h*(*SID_j_*║*r_j_*). Then *h*(*SID_j_*║*r_j_*) and *SID_j_* are stored on the *GWN*’s storage. The pre-configured data is transferred by using physical delivery.

### 3.3. Registration Phase

#### 3.3.1. Registration Phase for Users

Step 1: *U_i_* → *GWN*: {*X*_0_, *X*_1_, *REG_i_*, *t*_1_}
  *U_i_* chooses his/her identity *ID_i_*, password *PW_i_*, random numbers *r* and *r_i_*, and computes *K_UG_* = *T_r_*(*PK_G_*) mod *p*, *X*_0_ = *T_r_*(*x*) mod *p*, *REG_i_* = *K_UG_* ⨁ (*ID^pre^_i_*║*ID_i_*║*h*(*ID_i_*║*PW_i_*║*r_i_*), and *X*_1_ = *h*(*K_UG_*║*h*(*ID_i_*║*PW_i_*║*r_i_*)║*t*_1_), where *t*_1_ is the current timestamp. Then *U_i_* sends {*X_0_*, *X*_1_, *REG_i_*, *t*_1_} to *GWN*.
Step 2: *GWN* → *U_i_*: {*Y*_0_, *Y*_1_}
  Upon receiving the register message form *Ui*, *GWN* checks the validity of *t*1 and computes *K_UG_* = *T_K__GWN_*(*X*0) mod *p* and *ID^pre^_i_*║*ID_i_*║*h*(*ID_i_*║*PW_i_*║*r_i_*) = *REG_i_* ⨁ *K_UG_*, and extracts (*ID^pre^_i_*, *ID_i_*, *h*(*ID_i_*║*PW_i_*║*r_i_*)). If *GWN* successfully checks *h*(*K_UG_*║*h*(*ID_i_*║*PW_i_* ║*r_i_*)║*t*_1_) =? *X*_1_ and verifies that *ID^pre^_i_* is in *GWN*’s storage and has not been registered, then generates an expiration time *E_i_*, and computes *U_i_*’s temporal credential *TCR_i_*= *h*(*K_GMN_*||*ID_i_*||*E_i_*), *D*1= *TCR_i_*⨁ *h*(*ID_i_*║*PW_i_*║*r_i_*), *Y*_0_ = *D*_1_ ⨁ *h*(*K_UG_*║*t*_1_) and *Y*_1_ = *h*(*D*_1_║*K_UG_*║*t*_1_). Then, *GWN* sends {*Y*_0_, *Y*_1_} to *U_i_*. *GWN* also stores (*h*(*ID_i_*), *E_i_*) in its storage and maintains a status-bit *b* and a last login field to indicate the status of the user. If *U_i_* logins *GWN*, *b* = 1, otherwise *b* = 0.
Step 3: After receiving the response message form *GWN*, *U_i_* computes *D*_1_ = *Y*_0_ ⨁ *h*(*K_UG_*║*t*_1_), checks *h*(*D*_1_║*K_UG_*║*t*_1_) =? *Y*_1_. If successful, *U_i_* inserts (*D*_1_, *PK_G_*, *T*(.), *x* , *p*, *h*(.), *r_i_*) into a smartcard and finishes the registration.

#### 3.3.2. Registration Phase for Sensor Nodes

Step 1: *S_j_* → *GWN*: {*SID_j_*, *Z*_0_, *t*_2_}
  *S_j_* computes *REG_j_* = *h*(*SID_j_*║*r_j_*), *Z*_0_ = *h*(*REG_j_*║*t*_2_), and sends {*SID_j_*, *Z*_0_, *t*_2_} to *GWN*, where *t*_2_ is the current timestamp.
Step 2: *GWN* → *S_j_*: {*SID_j_*, *Y*_2_, *Y*_3_}
  Upon receiving {*SID_j_*, *Z*_0_, *t*_2_}, *GWN* successfully checks the validity of *t*_2_ and *h*(*REG_j_*||*t*_2_) =? *Z*_0_ and verifies that *SID_j_* has not been registered, then computes *S_j_*’s temporal credential *TCR_j_* = *h*(*K_GWN_*║*REG_j_*), *Q_j_* = *TCR_j_* ⨁ *REG_j_*, *Y*_2_ = *TCR_j_* ⨁ *h*(*t*_2_║*REG_j_*), *Y*_3_ = *h*(*TCR_j_*║*REG_j_*║*t*_2_) stores (*SID_j_*, *Q_j_*) in its storage, and sends {*SID_j_*, *Y*_2_, *Y*_3_} to *S_j_*.
Step 3: *S_j_* computes its temporal credential *TCR_j_* = *Y*_2_ ⨁ *h*(*t*_2_║*REG_j_*), checks *h*(*TCR_j_*║*REG_j_*║*t*_2_) =? *Y*_3_, and stores (*SID_j_*, *TCR_j_*, *REG_j_*, *T*(.), *x*, *p*, *h*(.)) in its storage.

### 3.4. Login and Authentication Phase

**Figure 1:**
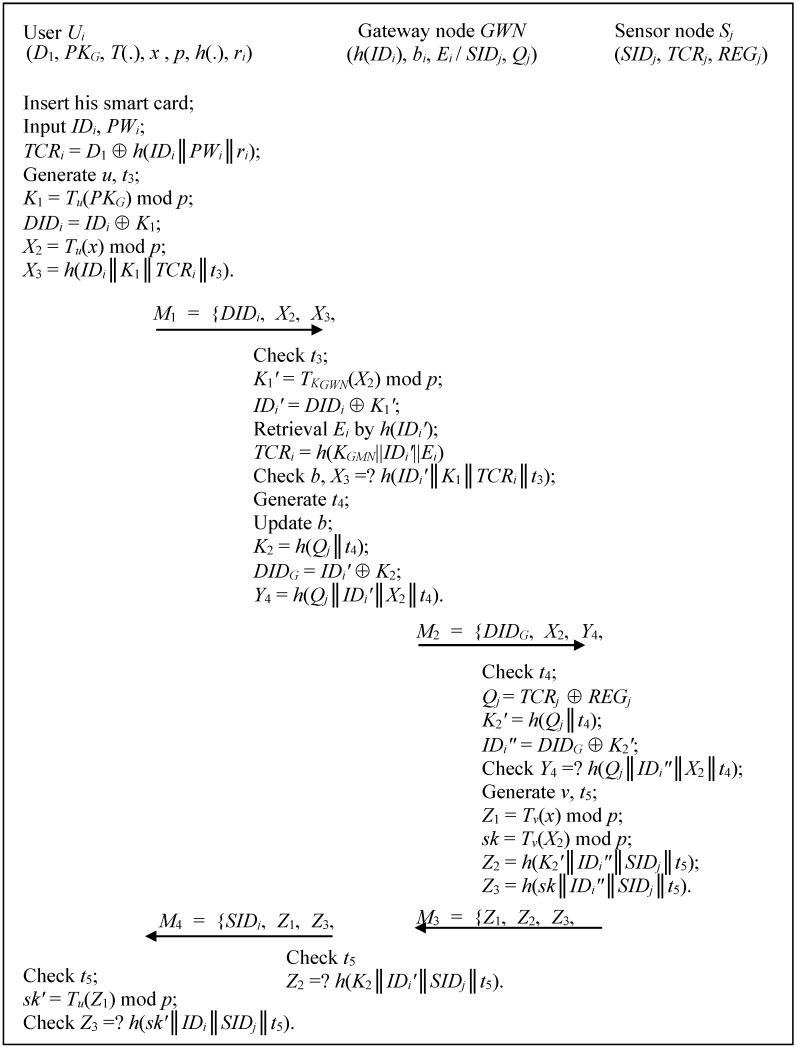
The login and authentication phase of the proposed scheme for WSNs.

In this phase, as shown in Figure 1, *U_i_* and *GWN* authenticate each other by performing the following steps:

Step 1: *U_i_* → *GWN*: *M*_1_ = {*DID_i_*, *X*_2_, *X*_3_, *t*_3_}
  *U_i_* inserts his smart card, inputs *ID_i_*, and *PW_i_*, computes *TCR_i_* = *D*_1_ ⨁ *h*(*ID_i_*║*PW_i_*║*r_i_*), generates a random number *u*, calculates *K*_1_ = *T_u_*(*PK_G_*) mod *p*, *DID_i_* = *ID_i_* ⨁ *K*_1_ and *X*_2_ = *T_u_*(*x*) mod *p*, *X*_3_ = *h*(*ID_i_*║*K*_1_║*TCR_i_*║*t*_3_), where *t*_3_ is the current timestamp, and sends *M*_1_ = {*DID_i_*, *X*_2_, *X*_3_, *t*_3_} to *GWN*.

Step 2: *GWN* → *S_j_*: *M*_2_ = {*DID_G_*, *X*_2_, *Y*_4_, *t*_4_}
  Upon receiving *M*_1_, *GWN* checks the validity of *t*_3_. If unsuccessful, *GWN* rejects this service request; Otherwise *GWN* computes *K*_1_*'* = *T_K__GWN_*(*X*_2_) mod *p*, *ID_i_'* = *DID_i_* ⨁ *K*_1_*'*, retrieval *E_i_* by *h*(*ID_i_'*), computes *TCR_i_* = *h*(*K_GMN_*||*ID_i_'*||*E_i_*), and checks the status-bit, *X*_3_ =? *h*(*ID_i_'*║*K*_1_║*TCR_i_*║*t*_3_). If unsuccessful, *GWN* rejects this service request; Otherwise *GWN* updates the status-bit, and chooses an accessed sensor node sensor node *S_j_* which is nearby and suitable, computes *K*_2_ = *h*(*Q_j_*║*t*_4_), *DID_G_* = *ID_i_'* ⨁ *K*_2_, *Y*_4_ = *h*(*Q_j_*║*ID_i_'*║*X*_2_║*t*_4_), where *t*_4_ is the current timestamp, and sends *M*_2_ = {*DID_G_*, *X*_2_, *Y*_4_, *t*_4_} to *S_j_*.
Step 3: *S_j_* → *GWN*: *M*_3_ = {*Z*_1_, *Z*_2_, *Z*_3_, *t*_5_}
  Upon receiving *M*_2_, *S_j_* checks the validity of *t*_4_. If unsuccessful, *S_j_* aborts this service request; Otherwise *S_j_* computes *Q_j_* = *TCR_j_* ⨁ *REG_j_*, *K*_2_*'* = *h*(*Q_j_*║*t*_4_), *ID_i_"* = *DID_G_* ⨁ *K*_2_*'*, and checks *Y*_4_ =? *h*(*Q_j_*║*ID_i_"*║*X*_2_║*t*_4_). If unsuccessful, *S_j_* still aborts this service request; Otherwise, *S_j_* generates *v*, calculates *Z*_1_ = *T_v_*(*x*) mod *p*, *sk* = *T_v_*(*X*_2_) mod *p*, *Z*_2_ = *h*(*K*_2_*'*║*ID_i_"*║*SID_j_*║*t*_5_), *Z*_3_ = *h*(*sk*║*ID_i_"*║*SID_j_*║*t*_5_), where *t*_5_ is the current timestamp, and sends *M*_3_ = {*Z*_1_, *Z*_2_, *Z*_3_, *t*_5_} to *GWN*.
Step 4: *GWN* → *U_i_*: *M*_4_ = {*SID_j_*, *Z*_1_, *Z*_3_, *t*_5_}
  Upon receiving *M*_3_, *GWN* checks the validity of *t*_5_. If unsuccessful, *GWN* rejects this request; Otherwise, *GWN* authenticates *S_j_* by checking *Z*_2_ =? *h*(*K*_2_║*ID_i_'*║*SID_j_*║*t*_5_), and sends *M*_4_ = {*SID_j_*, *Z*_1_, *Z*_3_, *t*_5_} to *U_i_*.
Step 5: Upon receiving *M*_4_, *U_i_* checks the validity of *t*_5_. If unsuccessful, *U_i_* aborts this request; Otherwise, *U_i_* computes *sk'* = *T_u_*(*Z*_1_) mod *p* and authenticates *GWN* and *S_j_* by checking *Z*_3_ =? *h*(*sk'*║*ID_i_*║*SID_j_*║*t*_5_). Finally, *U_i_* and *S_j_* obtain a common session key *sk* = *T_uv_*(*x*) mod *p* for later securing communications.

### 3.5. Password Change Phase

A user *U_i_* changes his/her password by performing the following steps:

Step 1: *U_i_* inserts his smart card and inputs his/her identity *ID_i_*, old password *PW_i_*, and a new password *PW_i_'*.
Step 2: The smart card computes *Q_i_* = *h*(*ID_i_*║*PW_i_*║*r_i_*) and *Q_i_'* = *h*(*ID_i_*║*PW_i_'*║*r_i_*) and *D*_1_*'* = *D*_1_ ⨁ *Q_i_* ⨁ *Q_i_'*. Then the smart card replaces *D*_1_ with *D*_1_*'*.

## 4. Security Analyses

This section analyzes the security of the proposed authenticated key agreement scheme, which provides mutual authentication, session key security and privacy protection for users, and resists potential attacks, including privileged insider attacks, password guessing attacks, impersonation attacks, stolen verifier attacks and many-logged-in-users attacks. The details are described below.

### 4.1. Communication Model

#### 4.1.1. Communicating Participants: 

The proposed scheme involves a user *U_i_*, a sensor node *S_j_*, and a gateway node *GWN*. *U_i_* and *S_j_* authenticate each other and establish a common session key *sk* with the help of the *GWN*. A participant may be involved in several instances, called oracles, of distinct concurrent executions of the proposed scheme **P**. The instance *m* of participant *V* is denoted as Π*_V_^m^*. 

#### 4.1.2. Oracle Queries: 

Oracle queries model the capabilities of adversary A, and are described below:

(1) ***Send***(Π*_V_^m^*, *M*): This query models the capacity of an adversary A to control all communications in **P**. A sends a message *M* to oracle Π*_V_^m^*; then Π*_V_^m^* sends back a response message using **P**. A can initiate the execution of **P** by sending a query (Π*_V_^m^*, "*start*") to a user oracle Π*_V_^m^*.
(2) ***Corrupt***(*V*): This query models the perfect forward secrecy of **P**, meaning that a compromised long-lived key fails to endanger previous session keys. The adversary A sends a corrupt query to a participant *V*, and returns *V*'s long-life key.
(3) ***Hash***(*M*): This query models adversary A’s reception of hash results by sending queries to a random oracle Ω. Upon receiving a query, Ωchecks whether a record (*M*, *r*) has been queried and recorded in the **H***-table*. If (*M*, *r*) in the **H***-table*, then Ω replies *r* to A; otherwise it returns a nonce *r'*, and keeps (*M*, *r'*) in the **H***-table*.
(4) ***Reveal***(Π*_V_^m^*): This query models the known key security of **P**: a compromised session key fails to reveal other session keys, and is only available if oracle Π*_V_^m^* has accepted.
(5) ***Test***(Π*_V_^m^*): This query models the session key security to determine the indistinguishability of the real session key from a random string. During the execution of scheme **P**, adversary A sends queries to the oracle, including a single ***Test*** query at any time. Then, Π*_V_^m^* flips an unbiased coin *c*. If *c* equals 1, then Π*_V_^m^* returns the real session key *sk*; otherwise, it returns a random string to A.

### 4.2. Security Definitions

#### 4.2.1. Partnering: Two user oracles Π*_U__i_^m^* and Π*_S__j_^n^* are partnered if:

(1) Π*_U__i_^m^* and Π*_S__j_^n^* directly exchange message flows and
(2) only Π*_U__i_^m^* and Π*_S__j_^n^* have the same session key *sk*.

#### 4.2.2. Freshness: An Oracle Π*_U__i_^m^* is **Fresh** in **P** if:

(1) Π*_U__i_^m^* or Π*_S__j_^n^* has accepted a session key *sk* and
(2) Π*_U__i_^m^* and Π*_S__j_^n^* have not been sent a **Reveal** query.

#### 4.2.3. Session Key Security (AKE Security): 

This definition allows an adversary to generate many ***Test*** queries. If a ***Test*** query is generated concerning a client instance that has not *accepted*, then the invalid symbol ⊥ is returned. If a ***Test*** query is generated concerning an instance of an honest participant whose intended partner is dishonest or an instance of a dishonest participant, then replies with the real session key. Otherwise, the reply to the ***Test*** query provides either the real session key or a random string, as determine by flipping an unbiased coin, *c*. The adversary seeks to guess correctly the value of the hidden bit *c* that is used by the Test oracle. The *ake-advantage* of the event that an adversary violates the indistinguishability of scheme **P** is denoted as *Adv_P_^ake^*(A)*.* The scheme **P** is AKE-secure if *Adv_P_^ake^*(A) is negligible [30,31,32].

#### 4.2.4. Mutual Authentication (MA Security)

In the execution of **P**, the adversary A violates mutual authentication if A can fake the authenticator. The probability of this event is denoted by *Adv_P_^ma^*(A). The scheme **P** is MA-secure if *Adv_P_^ma^*(A) is negligible [33].

### 4.3. Providing Session Key Security (AKE Security)

The following lemma describes the Difference Lemma, which is made used within our sequence of games [34].

**Lemma 1 (Difference Lemma).** *Let A, B and F be events defined in some probability distribution, and suppose that A∧¬F⟺ B∧¬F. Then*

|*Pr[A] − Pr[B]| ≤ Pr[F]*

*The following theorem shows that the proposed scheme involving U_i_ and S_j_ has AKE security if the used hash function is secure and the extended chaotic map-based Diffie-Hellman assumption holds*.

**Theorem 1.** *Let Adv^ecmdh^ be the advantage that an ECMDH attacker solves the extended chaotic map-based Diffie-Hellman problem within time t. Then, the probability that an adversary breaks the AKE security of the proposed scheme:*

*Adv_P_^ake^*(*t'*, *q_exe_*, *q_test_*, *q_se_*, *q_ake_*) ≤ 2⋅*Adv^ecmdh^*(*t*, *q_test_*, *q_se_*, *q_ake_*)

*within time t' and t'* ≤ *t* +4(*q_exe_*+*q_ake_*)*τ, where q_exe_ denotes the number of queries to the Execute oracle; q_test_ denotes the number of queries to the Test oracle; q_se_ denotes the numbers of the Send queries; q_ake_ denotes the number of queries to the final AKE scheme; and τ is the time to perform an extended chaotic map operation.*

**Proof of Theorem 1.** Each game G*_i_* defines the probability of the event *E_i_* that the adversary wins this game. The first game G_0_ is the real attack against the proposed scheme and the final game G_2_ concludes that the adversary has a negligible advantage to break the AKE security of the proposed scheme:

**Game** G_0_: This game corresponds to the real attack. By definition, we have

*Adv_P_^ake^*(A) = |2Pr[*E*_0_] − 1| (5)

**Game** G_1_: This game simulates all oracles as in previous game except for modifying the simulation of ***Send*** queries refereeing the flows containing *T_u_*(*x*) mod *p* and *T_v_*(*x*) mod *p* of the proposed scheme, and the simulation of the **Test**(Π*_V_^m^*) oracle to avoid relying on the knowledge of *u*, *v* and *w* used to compute the answer to these queries. Assume that (*X*, *Y*, *Z*) = (*T_u_*(*x*) mod *p*, *T_v_*(*x*) mod *p*, *T_u⋅v_*(*x*) mod *p*) is a random extended chaotic map-based Diffie-Hellman triple. A simulator Σ simulates the oracles for all sessions by using this triple (*X*, *Y*, *Z*) and the classical random self-reducibility of the extended chaotic map-based Diffie-Hellman problem. Next, Σ sets up all parameters and secret keys of the scheme, and picks a random number *m* ∈ [1, *q_se_*] and answers the oracle queries according to the proposed scheme. Σ thus can correctly return the *Test* queries. Additionally, the random variables in G_0_ is replaced by another random variables in G_1_. Then we have that G_0_ and G_1_ is equivalent, and thus:

Pr[*E*_0_] = Pr[*E*_1_] (6)

Game G_2_: This game simulates all oracles as in previous game except that all rules are computed using a triple (*X*, *Y*, *Z*) from a random distribution (*T_u_*(*x*) mod *p*, *T_v_*(*x*) mod *p*, *T_w_*(*x*) mod *p*), instead of an extended chaotic map-based Diffie-Hellman triple. Let a challenger A_ecdh_ try to violate the indistinguishability of the extended chaotic map-based Diffie-Hellman problem; and an adversary A_ake_ be constructed to break the session key security. A_ecdh_ returns the real session key *sk* (if *c* = 1) or a random string (otherwise) to A_ake_ by flipping an unbiased coin *c* ∈ {0,1}. Then A_ake_ wins the game if its output bit *c'* equals *c*. A_ecmdh_ is asked *Send*, *Corrupt* or *Test* queries, and returns the responses by using a previous experiment except for (*X*, *Y*, *Z*) that it had received as input. If A_ake_ outputs *c*, then A_ecmdh_ outputs 1; otherwise, A_ecmdh_ outputs 0. If (*X*, *Y*, *Z*) is a real extended chaotic map-based Diffie-Hellman triple, then A_ecmdh_ runs A_ake_ in G_1_ and thus the probability of the event that A_ecmdh_ outputs 1 equals the probability of *E*_1_. If (*X*, *Y*, *Z*) is a random triple, A_ecmdh_ runs A_ake_ in G_2_ and thus the probability of the event that A_ecdh_ outputs 1 equals the probability of *E*_2_. Therefore, we have:

|Pr[*E*_1_] − Pr[*E*_2_]|≤*Adv^ecmdh^*(A_ecmdh_) (7)

Since the coin bit *c* and all sessions keys are random and independent, we have

Pr[*E*_2_] = 1/2 (8)

By combining Equations (5)–(8) and using Lemma 1, we have:

*Adv_P_^ake^*(A_ake_) ≤ 2⋅*Adv^ecmdh^*(A_ecmdh_)

Then the proof is concluded.

### 4.4. Providing Mutual Authentication

The following theorem shows that the proposed scheme has MA security if the used hash function is secure and the proposed scheme has AKE security:

**Theorem 2.** *Let Adv_P_^ake^ denote the advantage that an adversary breaks the AKE security of the proposed scheme, and Adv_P_^ma^ denote the advantage that an adversary violates the mutual authentication of the proposed scheme. Then:*

*Adv_P_^ma^*(*t"*, *q_se_*, *q_h_*) ≤ 2⋅*Adv_P_^ake^*(*t'*, *q_se_*, *q_h_*) + *q_h_*^2^_/2^*l*−1^_

*within time t" and t"* ≤ *t'* + (*q_se_*+ *q_h_*)⋅*t_relay_* + 2⋅*τ*, *where q_h_ denotes the numbers of the Hash queries; t_relay_ denotes the time to relay a query; l denotes the security parameter and the parameters q_se_, t' and τ are defined as in Theorems 1.*

**Proof of Theorem 2.** The start game G*^ma^*_0_ is the real attack against the proposed scheme and the final game G*^ma^*_2_ concludes that the adversary has a negligible advantage to break MA security of the proposed scheme. The challenger A_1_ attempts to break AKE security of the proposed scheme and the adversary A_ma_ is constructed to break MA security of the proposed scheme. The adversary A_ma_ wins this game if he successfully fakes the authenticator:

**Game** G*^ma^*_0_: This game corresponds to the real attack. By definition, we have:

*Adv_P_^ma^*(A_ma_)=|2Pr[*E*_0_] − 1| (9)

**Game** G*^ma^*_1_: This game simulates all oracles as in previous game except for using a table list **H** to simulate *Hash* queries involving *U_i_* and *GWN*, and involving *GWN* and *S_j_*. Then, games G*^ma^*_0_ and G*^ma^*_1_ are **undistinguishable** except collisions of **H***-table* in G*^ma^*_1_. By using the birthday paradox and Lemma 1, we have:

|Pr[*E*_0_] − Pr[*E*_1_]|≤ *q_h_*^2^_/2*^l^*_ (10)

where A_ma_ makes *q_h_* ***Hash*** queries involving *U_i_* and *GWN*, and involving *GWN* and *S_j_*. 

**Game** G*^ma^*_2_: This game simulates all oracles as in previous game except for replacing the session key *sk* with a random number. Then, A_ma_ is used for building an adversary A_1_ against the AKE security of the proposed scheme. Next, A_1_ arranges the parameters, simulates the proposed scheme and replies the oracle queries made by A_ma_ by using following scenarios.

— When receiving ***Send*** or ***Hash*** queries involving *U_i_* and *GWN*, and involving *GWN* and *S_j_*, A_1_ replies the results by executing the proposed scheme.
— When receiving ***Hash*** queries involving *U_i_* and *S_j_*, A_1_ replies corresponding authenticators to A_ma_ by making the same queries to the oracle Hash involving *U_i_* and *S_j_*.
— When receiving ***Test*** queries, A_1_ replies these queries by using the coin bit *c* that it has previously selected and the computed session keys.

Therefore, the probability of the event that A_1_ outputs 1 when the authenticator is obtained by the real session key equals the probability of the event that A_ma_ correctly guesses the hidden bit *c* in game G*^ma^*_1_. Similarly, the probability that A_1_ outputs 1 when the authenticator obtained by a random string equals the probability that A_ma_ correctly guesses the hidden bit *c* in game G*^ma^*_2_. Thus, by Lemma 1, we have:

|Pr[*E*_1_] − Pr[*E*_2_]| ≤ *Adv_P_^ake^*(A_1_) (11)

Since no information on the authenticator is leaked to the adversary, we have

Pr[*E*_2_] = 1/2 (12)

Combining Equations (9)–(12) and using Lemma 1, we have

*Adv_P_^ma^*(A_ma_) ≤ 2 *Adv_P_^ake^*(A_1_)+ *q_h_*^2^_/2^*l*−1^_

Then the proof is concluded.

### 4.5. Protecting Privacy of Users

**Theorem 3.** *The proposed scheme protects the privacy of users.*

**Proof of Theorem 3.** The proposed scheme protects user *U_i_*’s identity *ID_i_* using the temporary secret key *K*_1_ of the user and the gateway node, and enables any two request messages *M*_1_ = {*DID_i_*, *X*_2_, *X*_3_, *t*_3_} and *M*_1_*'* = {*DID_i_'*, *X*_2_*'*, *X*_3_*'*, *t*_3_*'*} from user *U_i_* to be independent and difficult to distinguish from each other, where *K*_1_ = *T_u_*(*PK_G_*) mod *p*, *DID_i_* = *ID_i_* ⨁ *K*_1_, *X*_2_ = *T_u_*(*x*) mod *p*, *X*_3_ = *h*(*ID_i_*║*K*_1_║*TCR_i_*║*t*_3_), *u* is a random number and *t*_3_ is a timestamp; and *K*_1_*'* = *T_u'_*(*PK_G_*) mod *p*, *DID_i_'* = *ID_i_* ⨁ *K*_1_*'*, *X*_2_*'* = *T_u'_*(*x*) mod *p*, *X*_3_*'* = *h*(*ID_i_*║*K*_1_*'*║*TCR_i_*║*t*_3_*'*), *u'* is a random number and *t*_3_*'* is a timestamp. The proposed scheme provides user anonymity and data unlinkability, and thus exhibits untrackability [29]. Accordingly, the privacy of users is protected.

### 4.6. Resistance to Privileged Insider Attacks 

**Theorem 4.** *The proposed scheme withstands privileged insider attacks.*

**Proof of Theorem 4.** In the registration phase, the user sends *REG_i_* rather than (*ID_i_*, *PW_i_*) to *GWN*, where *REG_i_* = *K_UG_* ⨁ (*ID^pre^_i_*║*ID_i_*║*h*(*ID_i_*║*PW_i_*║*r_i_*), *U_i_*’s identity *ID_i_* and password *PW_i_* are protected by a random number *r_i_*. Therefore, the privileged insider fails to obtain (*ID_i_*, *PW_i_*) and *REG_i_*, and fails correctly to compute *TCR_i_* = *D*_1_ ⨁ *h*(*ID_i_*║*PW_i_*║*r_i_*) (or *h*(*K_GMN_*||*ID_i_*||*E_i_*)), so the proposed scheme withstands the privileged insider attack.

### 4.7. Resistance to Impersonation Attacks

**Theorem 5.** *The proposed scheme withstands impersonation attacks.*

**Proof of Theorem 5.** An adversary who tries to impersonate *U_i_* fails to compute *TCR_i_* = *D*_1_ ⨁ *h*(*ID_i_*║*PW_i_*║*r_i_*) and *X*_3_ = *h*(*ID_i_*║*K*_1_║*TCR_i_*║*t*_3_), and cannot send out the correct request messages *M*_1_ = {*DID_i_*, *X*_2_, *X*_3_, *t*_3_} in the login and authentication phase without the correct *ID_i_*, *PW_i_* and (*D*_1_, *r_i_*) in *U_i_*’s smart card, where *t*_3_ is the timestamp. A failed login is detected by the *GWN* in Step 2 of the login and authentication phase, so the proposed scheme withstands impersonation attacks.

### 4.8. Resistance to Off-Line Password Guessing Attacks

**Theorem 6.** *The proposed scheme withstands off-line password guessing attacks.*

**Proof of Theorem 6.** In the proposed scheme, since reveal messages *M*_1_ = {*DID_i_*, *X*_2_, *X*_3_, *t*_3_}, *M*_2_ = {*DID_G_*, *X*_2_, *Y*_4_, *t*_4_}, *M*_3_ = {*Z*_1_, *Z*_2_, *Z*_3_, *t*_5_} and *M*_4_ = {*SID_j_*, *Z*_1_, *Z*_3_, *t*_5_} do not provide information about users’ passwords *PW_i_*, an adversary cannot confirm the accuracy of the passwords that have been guessed from *M*_1_, *M*_2_, *M*_3_ and *M*_4_, where *DID_i_* = *ID_i_* ⨁ *K*_1_, *K*_1_ = *T_u_*(*PK_G_*) mod *p*, *X*_2_ = *T_u_*(*x*) mod *p*, *X*_3_ = *h*(*ID_i_*║*K*_1_║*TCR_i_*║*t*_3_) and *TCR_i_* = *h*(*K_GMN_*||*ID_i_*||*E_i_*); *DID_G_* = *ID_i_'* ⨁ *K*_2_, *K*_2_ = *h*(*Q_j_*║*t*_4_) and *Y*_4_ = *h*(*Q_j_*║*ID_i_'*║*X*_2_║*t*_4_); and *Z*_1_ = *T_v_*(*x*) mod *p*, *Z*_2_ = *h*(*K*_2_*'*║*ID_i_"*║*SID_j_*║*t*_5_), *Z*_3_ = *h*(*sk*║*ID_i_"*║*SID_j_*║*t*_5_) and *sk* = *T_v_*(*X*_2_) mod *p*. Thus, off-line password guessing attacks are ineffective against the proposed scheme. 

### 4.9. Resistance to Undetectable On-Line Password Guessing Attacks

**Theorem 7.** *The proposed scheme withstands on-line password guessing attacks.*

**Proof of Theorem 7.** Again, the revealed messages *M*_1_, *M*_2_, *M*_3_ and *M*_4_ do not provide information about a user’s password *PW_i_*. Accordingly, an attacker has difficulty in guessing the password in an on-line transaction, and the scheme thus resists undetectable on-line password guessing attacks.

### 4.10. Resistance to Stolen Verifier Attacks

**Theorem 8.** *The proposed scheme withstands stolen verifier attacks.*

**Proof of Theorem 8.** In the proposed scheme, the *GWN* keeps (*h*(*ID_i_*), *E_i_*) in the verifier table for each user *U_i_*. An adversary who steals the *GWN*’s verifier table and copies (*h*(*ID_i_*), *E_i_*) still fails to compute *TCR_i_* = *D*_1_ ⨁ *h*(*ID_i_*║*PW_i_*║*r_i_*), *DID_i_* = *ID_i_* ⨁ *K*_1_ and *X*_3_ = *h*(*ID_i_*║*K*_1_║*TCR_i_*║*t*_3_) without knowledge of user *U_i_*’s *ID_i_*, *PW_i_*, *r_i_* and *D*_1_, where *u* is a random number, *K*_1_ = *T_u_*(*PK_G_*) mod *p*, *X*_2_ = *T_u_*(*x*) mod *p* and *t*_3_ is the timestamp. The adversary fails to send out *M*_1_ = {*DID_i_*, *X*_2_, *X*_3_, *t*_3_} in Step 1, and a failed login is detected by the *GWN*. Therefore, the proposed scheme resists stolen verifier attacks.

### 4.11. Resistance to Lost Smartcard Attacks

**Theorem 9.** *The proposed scheme withstands lost smartcard attacks.*

**Proof of Theorem 9.** An adversary who steals user *U_i_*’s smartcard and copies the message (*D*_1_, *PK_G_*, *T*(.), *x*, *p*, *h*(.), *r_i_*) still fails to compute *TCR_i_* = *D*_1_ ⨁ *h*(*ID_i_*║*PW_i_*║*r_i_*) and *X*_3_ = *h*(*ID_i_*║*K*_1_║*TCR_i_*║*t*_3_), where *t*_3_ is the timestamp, and so cannot send out the correct messages *M*_1_ = {*DID_i_*, *X*_2_, *X*_3_, *t*_3_} in Step 1 of the login and authentication phase without the correct *ID_i_* and *PW_i_*. The *GWN* will detect a failed login Step 2 of the login and authentication phase, so the proposed scheme withstands lost smartcard attacks.

### 4.12. Resistance to Many Logged-in Users Attacks

**Theorem 10.** *The proposed scheme withstands many-logged-in-users attacks.*

**Proof of Theorem 10.** Assume that *U_i_*’s login information (*ID_i_*, *PW_i_*, *T*(.), *x*, *p*, *h*(.), *r_i_*) is leaked to more than one non-registered user. The *GWN* also maintains a status-bit field and a last login field in its verifier table to prevent simultaneous duplicate logins. Therefore, the proposed scheme withstands many-logged-in-users attacks.

## 5. Performance Analyses and Functionality Comparisons

### 5.1. Performance Analyses

Table 2 compares the performance of the proposed scheme with those of the schemes developed by Yeh *et al*. [16], Xue *et al*. [8], Li *et al*. [9] and Kim *et al*. [35], where *T_h_* is the execution time for a one-way hash operation; *T_c_* is the execution time for a Chebyshev chaotic map operation, and *T_e_* is the execution time for a scalar multiplication operation on an elliptic curve.

The first comparison made concerns the computational cost for user *U_i_*, sensor node *S_j_* and the gateway node *GWN*. The scheme of Yeh *et al*., [16] employs encryptions and decryptions on an elliptic curve, and has a greater computational cost than related schemes [8,9,35], which use only hash operations. Since *T_c_* approximates *T_h_*, where *T_h_* is obtained by using the hash functions SHA-1 and MD5 [36,37,38], the proposed scheme requires six chaotic map operations and 13 hash function operations and so has a low computational burden.

**Table 2:** The performance comparisons of the related schemes and the proposed scheme.

		Yeh *et al.* [16]	Xue *et al.* [8]	Li *et al.* [9]	Kim *et al.* [35]	Our Scheme
	*U_i_*	2 *T_e_* + 1 *T_h_*	7 *T_h_*	9 *T_h_*	8 *T_h_*	3 *T_c_* + 3 *T_h_*
Computations	*S_j_*	2 *T_e_* + 3 *T_h_*	5 *T_h_*	6 *T_h_*	2 *T_h_*	2 *T_c_* + 4 *T_h_*
	*GWN*	4 *T_e_* + 4 *T_h_*	10 *T_h_*	11 *T_h_*	8 *T_h_*	1 *T_c_* + 6 *T_h_*
	Total	8 *T_e_* + 8 *T_h_*	22 *T_h_*	26 *T_h_*	18 *T_h_*	6 *T_c_* + 13 *T_h_*

### 5.2. Functionality Comparisons

Table 3 compares the proposed scheme and related schemes in terms of functionality, and specifically the meeting of security requirements and resistance to possible attacks. The schemes that were developed by Yeh *et al.*, Xue *et al.*, Li *et al.* and Kim *et al.* all fail to protect users’ privacy. Additionally, the scheme of Yeh *et al.* fails to withstand password guessing, lost smart card and many-logged-in-users attacks. The scheme of Xue *et al.* fails to withstand privileged insider, password guessing, stolen verifier, lost smart card and many-logged-in-users attacks. The scheme of Li *et al.* fails to withstand impersonation and stolen verifier attacks. Only the proposed scheme withstands all possible attacks and protects privacy. Thus, the proposed scheme provides greater functionality; exhibits more favorable security-related properties, and has a lower computational cost than the other schemes.

**Table 3:** The functionality comparisons of the related schemes and the proposed scheme.

	Yeh *et al.* [16]	Xue *et al.* [8]	Li *et al.* [9]	Kim *et al.* [3,5]	Our Scheme
Providing mutual authentication	Yes	Yes	Yes	Yes	Yes
Providing session key security	Yes	Yes	Yes	Yes	Yes
Providing privacy protection	No	No	No	No	Yes
Resisting privileged insider attacks	Yes	No	Yes	Yes	Yes
Resisting to impersonation attacks	Yes	Yes	No	Yes	Yes
Resisting password guessing attacks	No	No	Yes	Yes	Yes
Resisting stolen verifier attacks	Yes	No	No	Yes	Yes
Resisting lost smartcard attacks	No	No	Yes	Yes	Yes
Resisting many logged-in users attacks	No	No	Yes	Yes	Yes

## 6. Conclusions

This study addresses the weaknesses of the temporal credential-based authenticated key agreement scheme developed by Li *et al.*, which enables an adversary to impersonate legitimate users, to perform a stolen verifier attack to calculate all used session keys and transmitted secrets of users and sensor nodes, and to reveal users’ identities. A new temporal credential-based authenticated key agreement scheme that uses chaotic maps is developed for WSNs. The proposed scheme protects each user’s identity using a temporary secret key; conceals each user’s private parameters, and reduces the number of redundant parameters concerning the user’s identity and password in the verifier table of the *GWN*. Therefore, the proposed scheme does not have any of the weaknesses of previous schemes. Additionally, session key security is based on the extended chaotic maps-based Diffie-Hellman problem, and the proposed scheme thus exhibits perfect forward secrecy and known-key security. The proposed scheme not only eliminates the weaknesses of previous approaches, but also increases security and efficiency.
